# Integration of machine learning and experimental validation reveals new lipid-lowering drug candidates

**DOI:** 10.1038/s41401-025-01539-1

**Published:** 2025-04-15

**Authors:** Jing-hong Chen, Ke-xin Li, Chao-fan Fan, Hong Yang, Zhi-rou Zhang, Yi-han Chen, Chang Qi, Ang-hua Li, An-qi Lin, Xin Chen, Peng Luo

**Affiliations:** 1 Department of Oncology, Zhujiang Hospital, The Second School of Clinical Medicine, Southern Medical University, Guangzhou, 510280 China; 2 Donghai County People’s Hospital - Jiangnan University Smart Healthcare Joint Laboratory, Donghai County People’s Hospital (Affiliated Kangda College of Nanjing Medical University), Lianyungang, 222000 China; 3 Department of Pulmonary and Critical Care Medicine, Zhujiang Hospital, Southern Medical University, Guangzhou, 510280 China; 4 The University of Hong Kong, Hong Kong, China

**Keywords:** hyperlipidemia, machine learning, retrospective clinical data analysis, animal experimental validation, molecular docking and dynamics simulation

## Abstract

Hyperlipidemia, a major risk factor for cardiovascular diseases, is associated with limitations in clinical lipid-lowering medications. Drug repurposing strategies expedite the research process and mitigate development costs, offering an innovative approach to drug discovery. This study employed systematic literature and guidelines review to compile a training set comprising 176 lipid-lowering drugs and 3254 non-lipid-lowering drugs. Multiple machine learning models were developed to predict the lipid-lowering potential of drugs. A multi-tiered validation strategy was implemented, encompassing large-scale retrospective clinical data analysis, standardized animal studies, molecular docking simulations and dynamics analyses. Through a comprehensive screening analysis utilizing machine learning, 29 FDA-approved drugs with lipid-lowering potential were identified. Clinical data analysis confirmed that four candidate drugs, with Argatroban as the representative, demonstrated lipid-lowering effects. In animal experiments, the candidate drugs significantly improved multiple blood lipid parameters. Molecular docking and dynamics simulations elucidated the binding patterns and stability of candidate drugs in interaction with related targets. We successfully identified multiple non-lipid-lowering drugs with lipid-lowering potential by integrating state-of-the-art machine learning techniques with multi-level validation methods, thereby providing new insights into lipid-lowering drugs, establishing a paradigm for AI-based drug repositioning research, and expanding the repertoire of lipid-lowering medications available to clinicians.

## Introduction

Hyperlipidemia is a metabolic disorder characterized by abnormally elevated levels of plasma lipids and lipoproteins in the bloodstream. Based on the specific types of abnormal lipids and lipoproteins, hyperlipidemia can be further classified into four distinct subtypes: hypercholesterolemia, hypertriglyceridemia, mixed hyperlipidemia, and low high-density lipoprotein cholesterolemia [1, 2]. The diagnosis of hyperlipidemia is typically based on the assessment of several key indicators in blood tests, primarily comprising total cholesterol (TC), low-density lipoprotein cholesterol (LDL-C), high-density lipoprotein cholesterol (HDL-C), and triglycerides (TG) [3]. According to the American Heart Association’s 2023 Heart Disease and Stroke Statistics Update [4], the prevalence of hypercholesterolemia (defined as TC ≥ 200 mg/dL) among US adults was 34.70%, while the prevalence of elevated LDL-C levels (≥130 mg/dL) was 25.50% during the period from 2017 to 2020. Furthermore, it is noteworthy that the prevalence of hyperlipidemia among younger demographic groups has demonstrated a statistically significant upward trend in recent years [5]. In addition to the increasing prevalence trends elucidated by the aforementioned statistics, the clinical importance of hyperlipidemia as a principal risk factor for cardiovascular diseases warrants significant attention. A study emphasizes that the proportional relationship between cholesterol and IHD mortality decreases with age [6]. With respect to pathogenesis, hyperlipidemia predominantly elevates the risk of cardiovascular events through mechanisms including the acceleration of atherosclerosis and the promotion of plaque formation and rupture [7]. In conclusion, the dual impact of hyperlipidemia on epidemiology and cardiovascular health emphasizes the critical necessity for sustained vigilance and efficacious management of this condition.

Pharmacotherapy represents a crucial approach in treating hyperlipidemia, wherein lipid-lowering medications reduce blood lipid levels through various mechanisms, thus preventing and treating cardiovascular diseases. At present, commonly used lipid-lowering medications in clinical practice predominantly include statins, cholesterol absorption inhibitors, PCSK9 inhibitors, and so on [3]. Statins significantly reduce LDL-C levels by inhibiting HMG-CoA reductase, a key enzyme in cholesterol synthesis [8]. Cholesterol absorption inhibitors, exemplified by ezetimibe, further lower LDL-C levels when used in combination with statins by inhibiting intestinal cholesterol absorption [9]. PCSK9 inhibitors significantly reduce LDL-C levels by blocking the binding of PCSK9 to LDL receptors, consequently increasing LDL receptor recycling [10]. Despite the significant success of lipid-lowering medications in reducing blood lipids and preventing cardiovascular diseases, their application continues to face several challenges. Specifically, some patients demonstrate poor tolerance to existing lipid-lowering medications, potentially manifesting adverse reactions such as muscle symptoms and liver function abnormalities [11]. Moreover, some patients exhibit reduced sensitivity to certain lipid-lowering medications: even after receiving maximum-dose statin therapy, they continue to show inadequate therapeutic effects and fail to attain lipid control targets [12]. To address these challenges, the development of new lipid-lowering therapeutic strategies remains imperative, with the aim of providing patients with more effective treatment options.

In the pursuit of novel lipid-lowering therapeutic strategies, exploring new applications for existing non-lipid-lowering drugs presents a promising direction, potentially offering alternative treatment options for patients who are intolerant to or have developed resistance to traditional lipid-lowering medications. In recent years, several non-lipid-lowering drugs have demonstrated potential in reducing blood lipid levels. For instance, metformin, a commonly prescribed antidiabetic medication, not only improves insulin sensitivity but also potentially lowers blood lipid levels through the activation of the AMPK signaling pathway and inhibition of hepatic fatty acid synthesis [13]. Traditional approaches to developing new drug therapies, such as high-throughput screening [14] and structure-based drug design [15], are often associated with high costs, lengthy development times, and substantial risks of failure. The rapid advancement of modern bioinformatics and artificial intelligence (AI) technologies has led to the widespread application of AI methods, particularly machine learning and deep learning, in the medical field [16, 17]. These technologies are bringing transformative impacts to disease diagnosis, drug development, and precision medicine.

Machine learning, as a computational approach that employs data mining and algorithmic analysis for prediction, demonstrates exceptional potential for application in drug development, particularly in identifying novel indications for existing drugs. In comparison with conventional methods, machine learning algorithms can autonomously extract features, discern patterns from extensive biomedical datasets, and elucidate potential drug-disease associations, thereby facilitating the prediction of novel drug indications [18]. This approach offers a robust complement to conventional drug development processes, potentially expediting development timelines, mitigating costs, and enhancing success rates [19]. For example, Li et al. employed MAI-TargetFisher demonstrates how machine learning enhances drug development by combining AI-based and biophysical modeling methods to predict drug-protein interactions across the human proteome, achieving higher accuracy and coverage than traditional approaches in identifying potential drug targets [20]. Zeng et al. developed deepDR, a network-based deep learning framework for drug repositioning by integrating analyses of 10 heterogeneous networks (including drug-disease, drug-side-effect, drug-target and drug-drug networks), revealing that several FDA-approved drugs like risperidone and aripiprazole may exhibit therapeutic efficacy against Alzheimer’s disease, and methylphenidate and pergolide against Parkinson’s disease [21]. These studies collectively demonstrate the potential of machine learning to accelerate the development of drug, thereby providing crucial leads for developing novel therapeutic strategies. However, the application of machine learning in identifying potential new lipid-lowering indications from non-lipid-lowering drugs remains limited. Existing research predominantly focuses on exploring the mechanisms of action and predicting the efficacy of established lipid-lowering drugs. This phenomenon may arise from the challenges in obtaining lipid-related molecular and phenotypic data, as well as the current limited understanding of the lipid-lowering potential of non-lipid-lowering drugs. Therefore, there is a pressing need for targeted research to systematically explore new lipid-lowering indications of non-lipid-lowering drugs, with the objective of developing novel approaches for the prevention and treatment of hyperlipidemia. This study seeks to address this critical issue, aiming to bridge the research gap in this field.

Given the aforementioned research gaps, this study aims to systematically explore the lipid-lowering potential of non-lipid-lowering drugs through the application of machine learning approaches. Specifically, we will compile a series of lipid-lowering drugs in conjunction with non-lipid-lowering drugs approved by the FDA from public databases and literature, elucidate the physicochemical properties of these drugs, and incorporate multiple machine learning algorithms to develop a model capable of accurately predicting the lipid-lowering efficacy of non-lipid-lowering drugs. To comprehensively evaluate the performance and generalizability of the model, we will conduct retrospective clinical data validation and animal experiments. Through comprehensive analysis of the model’s predictions and actual observations, we aim to identify non-lipid-lowering drugs with lipid-lowering potential and preliminarily elucidate their lipid-lowering mechanisms via molecular docking and molecular dynamics simulations. Based on these anticipated research findings, we expect to promote the application of machine learning in drug repurposing, facilitating the efficient and economical discovery of potential lipid-lowering drugs, thereby providing more diverse treatment options for hyperlipidemia patients and potentially improving their prognosis.

## Materials and methods

### Drug data download and preprocessing

We systematically compiled a comprehensive list of clinically effective lipid-lowering drugs from seven authoritative guidelines, including the guideline of Lipid Management in Patients with Endocrine Disorders [22], Chinese Guidelines for Lipid Management (2023) [23], Chinese guidelines for the management of dyslipidemia in adults (2016) [24], ESC/EAS Guidelines for the Management of Dyslipidemias (2019) [25], NICE Guidelines on Lipid Modification [26], AHA/ACC Multisociety Guideline on the Management of Blood Cholesterol (2018) [3], and the Cholesterol Treatment Trialists’ Collaboration [27]. These clinically effective lipid-lowering drugs are defined as medications demonstrating the capacity to reduce blood lipid levels (TC, LDL, VLDL, TG) and/or increase HDL levels. Subsequently, we conducted a systematic PubMed search for relevant literature published between January 1, 2014, and January 31, 2024, utilizing the following search strategy: ((((hyperlipoproteinemia[MeSH Terms] OR hypercholesterolemia[MeSH Terms] OR hypercholesterolemic xanthomatotic OR hyper low-density lipoproteinemia) AND “drug”[tiab]) AND (“therapeutics”[MeSH Terms] OR “therapeutics”[All Fields] OR “therapies”[All Fields] OR “therapy”[MeSH Subheading] OR “therapy”[All Fields] OR “therapy s”[All Fields] OR “therapys”[All Fields])) AND ((“Clinical Trial”[Publication Type] OR “Randomized Controlled Trial”[Publication Type]) OR (“Animal Experimentation”[MeSH Terms] OR “Mice”[MeSH Terms]) OR (“Cell Line”[MeSH Terms] OR “Cell Line, Tumor”[MeSH Terms]) OR (“Review”[Publication Type] OR “Meta-Analysis”[Publication Type] OR “Systematic Review”[Publication Type]))). We extracted and analyzed information on drugs with lipid-lowering effects from the identified literature. Notably, drugs reported in clinical settings solely for alleviating complications of hyperlipidemia, without direct lipid-lowering effects, were excluded from our analysis. Ultimately, we compiled a comprehensive list of 176 drugs with demonstrated lipid-lowering effects from both the guidelines and literature reviews (Table S1).

To construct a comprehensive drug list for subsequent analysis, we categorized the FDA-approved drugs as follows: FDA-approved drugs with clinically proven lipid-lowering effects and/or the 176 drugs identified through our literature search were classified as positive drugs (Table S1), i.e., drugs known to have lipid-lowering effects (*n* = 176). The remaining drugs were categorized as negative drugs (*n* = 3254). Furthermore, we manually assessed and recorded the strength and reliability of evidence for the lipid-lowering effects of each drug, adhering to the principles of evidence-based medicine. Specifically, we implemented a hierarchical scoring system: positive drugs derived from systematic reviews, meta-analyses, or randomized controlled trials were assigned a score of 5 (highest reliability and evidence strength); those from cohort studies were assigned 4; those from case-control studies or case reports were assigned 3; those from clinical experience were assigned 2; those from animal, in vitro, or cellular studies were assigned 1; and negative drugs were uniformly assigned 0 (lowest reliability and evidence strength).

To acquire comprehensive structural information of drug molecules, we systematically extracted the Simplified Molecular Input Line Entry System (SMILES) representations, molecular formulas, and PubChem Compound Identification for both positive and negative drugs from three authoritative chemical and drug databases: PubChem (https://pubmed.ncbi.nlm.nih.gov/), ChemSpider [28], and DrugBank [29]. SMILES is a linear representation method for molecular structures based on graph theory and topological principles, which facilitates feature extraction and similarity calculations in subsequent machine learning algorithms. Drugs with identical SMILES codes were considered to be the same compound to avoid redundancy in our analysis.

### Physicochemical characterization and molecular fingerprint conversion of drugs

We employed the RDKit (https://www.rdkit.org/) in Python to extract molecular descriptor information for each drug from SMILES codes, thus quantifying molecular structural features. We integrated 23 molecular descriptors as input features for our machine learning models. These descriptors can be primarily classified into two main categories: physicochemical properties of molecules and molecular fingerprints. With respect to the physicochemical properties of molecules, we included 16 critical parameters, including molecular weight, heavy atom count, and number of hydrogen bond acceptors (Table S2). These parameters reflect various aspects of drug molecules, encompassing size, polarity, hydrophobicity, and conformational flexibility, which are crucial for characterizing key features of drug-target interactions. Furthermore, we incorporated 7 types of molecular fingerprints to delineate the structural features of drug molecules. Molecular fingerprints are techniques for digitally encoding molecular structures and can be utilized to quantify structural similarities between different molecules. We implemented the following molecular fingerprints: Molecular ACCess System (MACCS), Avalon, topological fingerprint, Extended-Connectivity Fingerprints (ECFP), Functional-Class Fingerprints (FCFP), Layered ECFP (LECFP), and Layered FCFP (LFCFP) (Table S3). Through the comprehensive application of these diverse molecular fingerprints, we can characterize the structural features of drug molecules from multiple perspectives, thus elucidating key structural elements responsible for the lipid-lowering effects of drugs.

### Machine learning model construction and evaluation

To optimize the machine learning model and mitigate potential confounding effects from irrelevant variables, we implemented a two-step feature selection process combining correlation analysis and LASSO regression. In the first step, we employed Spearman correlation analysis to compute the correlation coefficient between each feature and the lipid-lowering effect. Features with correlation coefficients exceeding 0.03 were selected as preliminary candidates, ensuring a manageable feature set of 300–500 for each drug. Subsequently, we applied LASSO regression to the training set for further feature refinement. The optimal regularization parameter λ was determined through 10-fold cross-validation to balance model complexity and performance. This cross-validation approach involved randomly partitioning the training data into 10 equal-sized subsets, with each subset serving as a validation set while the remaining data were used for training. The λ value that minimized the mean cross-validated error was selected, yielding the final feature set for subsequent machine learning analysis. This rigorous validation strategy helped ensure model robustness and mitigate potential overfitting issues. The choice of LASSO regression was motivated by its ability to perform both feature selection and regularization simultaneously, making it particularly suitable for high-dimensional data with potential multicollinearity.

We developed machine learning models based on the continuous variables. The continuous model quantified the likelihood of such effects using an evidence-based medicine grading scale (ranging from 0 to 5, with 5 indicating the highest level of reliability). Drugs with predicted scores of 1 or higher were classified as having potential lipid-lowering effects, while those with scores below 1 were deemed to lack such effects. Higher scores correlated with increased confidence in the drug’s lipid-lowering efficacy. We also implemented a data partitioning strategy where the entire dataset was randomly split into training (70%) and testing (30%) sets, maintaining balanced proportions of both positive and negative samples in each set. This stratified splitting ensures that both sets contain representative distributions of the data, thereby reducing potential bias and improving the model’s generalizability.

We implemented a total of 68 machine learning models, including Random Forest (RF) [30], Support Vector Machine (SVM) [31], Gradient Boosting Machine (GBM) [32], Elastic Net (Enet) [33], Generalized Linear Model Boost (glmBoost) [34], Stepwise Generalized Linear [35], Ridge Regression (RR) [36], Lasso Regression [37], Stepwise Regression (SR) [38], and various combinations thereof. The specific parameters for each model and various model combinations, are detailed in Supplementary Table S4. To mitigate model complexity and prevent overfitting, we applied variable selection and model combination techniques to specific models, including RF [30], GBM [32], and Lasso Regression [37]. We evaluated model performance using five metrics: Area under the curve (AUC), F1-score, recall, accuracy, and specificity. For subsequent analysis, we selected the top ten models based on their AUC performance.

### Screening of potential lipid-lowering drugs

Candidate potential lipid-lowering drugs were defined as those identified as having lipid-lowering effects in at least 8 out of the top 10 continuous variable models with the highest AUC values. The number of positive identifications for each drug across these models was tallied. All drugs identified as positive in at least one model underwent a secondary manual review to exclude those with previously reported lipid-lowering effects in the existing literature. It should be emphasized that not all potential lipid-lowering drugs predicted by machine learning models underwent clinical data validation or animal experimental verification. During the screening process for potential lipid-lowering drugs intended for subsequent clinical data validation, certain candidates were excluded due to the absence of usage records in the clinical information database. Non-lipid-lowering drugs predicted to have lipid-lowering potential by a minimum of 8 continuous machine learning models were ultimately selected as candidates for retrospective clinical data studies. For animal experimental validation, the selection was limited to non-lipid-lowering drugs predicted to have lipid-lowering potential by all 10 continuous variable machine learning models, as well as those that demonstrated lipid-lowering potential in local validation.

### Clinical data validation

We conducted a retrospective analysis of blood lipid profiles for patients who received the previously described model-predicted drug treatments at Zhujiang Hospital from June 19, 1998, to May 26, 2024. The analyzed parameters included TC, LDL-C, HDL-C, TG, and lipoprotein(a). For each patient, only their earliest drug administration record was included in the analysis. Patients were included only if they had documented medication records and underwent at least two blood lipid profile measurements - one before and one after drug administration. Only patients with complete lipid profile data both before and after drug administration were included in the final statistical analysis. In our analysis, we included both the mean values of patients’ lipid parameters before and after medication. Patients were excluded if they met any of the following criteria: concurrent use of any known lipid-lowering medications during the study period, missing or incomplete lipid profile data, major changes in other medications known to affect lipid metabolism during the study period, or participation in other clinical trials during the study period. This study was approved by the Ethics Committee of Zhujiang Hospital, Southern Medical University, and written informed consent was obtained from all participants.

### In vivo experimental validation

Due to limitations in drug availability, we conducted animal experiments on 16 drugs that were either computationally predicted to have lipid-lowering potential or demonstrated lipid-lowering efficacy in preliminary clinical data analyses. The selected drugs included Levoxyl, Argatroban, Sorafenib, Prasterone, Atazanavir Sulfate, Ketoconazole, Fenoprofen Calcium, Alpha-Tocopherol Acetate, Sulfaphenazole, Cedazuridine, Dicurin Procaine, Dimenhydrinate, Procarbazine Hydrochloride, Cupric Chloride, Regorafenib, and Promega. Detailed information on specific drug brands is provided in Supplementary Table S5.

All animal experimental procedures were reviewed and approved by the Institutional Animal Care and Use Committee of Zhujiang Hospital, Southern Medical University. Experiments were conducted in strict compliance with established animal welfare and ethical guidelines. Four-week-old male C57BL/6 mice of specific-pathogen-free grade were obtained from Guangzhou Yongnuo Biotechnology Co., Ltd. Prior to experimentation, mice underwent a one-week acclimation period in the animal facility of Yongnuo Biotechnology. The animal housing facility maintained a 12-h light/dark cycle, with ambient temperature ranging from 18 to 23 °C and relative humidity levels of 40%–60%. Mice were randomly assigned to experimental drug groups or control groups prior to drug administration. Control groups received intraperitoneal injections of phosphate-buffered saline as a vehicle control. Experimental groups received intraperitoneal injections of the respective drugs every 48 h, for a total of five doses. Drug dosages are detailed in Supplementary Table S5. Blood collection and subsequent serum separation were performed 24 h following the final drug administration. The blood collection procedure was as follows: under full anesthesia, blood was drawn via cardiac puncture. Collected blood samples were transferred to centrifuge tubes and allowed to clot for 60 min at room temperature (23 ± 2 °C). Samples were centrifuged at 3,000 rpm for 15 min at 4 °C. The supernatant (serum) was carefully aspirated and stored at −80 °C pending further analysis. Quantification of blood lipid levels was performed by Savior Biotechnology Co., Ltd. The following lipid profile parameters were analyzed: TC, TG, HDL-C, and LDL-C.

### Molecular docking and prediction of drug-ligand-receptor interactions

Molecular docking is a sophisticated computer-aided drug design method that simulates the binding mode of small drug molecules to large biomolecular targets, accurately predicting the binding affinity and conformation of small drug molecules, thus guiding the optimization and screening of lead compounds [39]. We systematically collected 12 protein targets that are known to be closely related to lipid metabolism and are targeted by common lipid-lowering drugs from Drugbank [29], encompassing various receptors, enzymes, and coagulation factors. The selected targets include 3-hydroxy-3-methylglutaryl-coenzyme A reductase (HMGR) [40], 5-hydroxytryptamine receptor 4 (5-HT4R) [41], 5-hydroxytryptamine receptor 2 C (HTR2C) [42], 5-hydroxytryptamine receptor 2 A (HTR2A) [43], 5-hydroxytryptamine receptor 2B (HTR2B) [44], Coagulation factor X (FX) [45], Liver carboxylesterase 1 (CES1) [46], Microsomal triglyceride transfer protein large subunit (MTP) [47], Prostaglandin G/H synthase 2 (COX-2) [48], Retinoic acid receptor alpha (RXRA) [49], Thyroid hormone receptor alpha (TRα) [50], and Thyroid hormone receptor beta (TRβ) [50]. The three-dimensional structures of the aforementioned target proteins were retrieved from the PDB database (Table S6). To ensure the selection of the most suitable protein structure for each target with multiple PDB IDs, we applied the following rigorous criteria: (1) Organism specificity: We prioritized protein structures from Homo sapiens to ensure maximal relevance to human physiological environments; (2) Resolution quality: We prioritized structures with lower Å values, indicative of higher resolution, to obtain more detailed and accurate protein structure information. To avoid potential structural deficiencies associated with excessively low Å values, we established a lower limit of 1 Å for the resolution; (3) Determination method: We favored structures determined by X-RAY DIFFRACTION to acquire higher quality and resolution protein structure data; (4) Ligand presence: Recognizing that crystallized ligands offer crucial information about protein active sites and functions, which facilitates subsequent drug design and optimization, we gave preference to protein structures containing original crystallized ligands.

The three-dimensional structural files of the drugs were obtained from the PubChem database. Seven candidate potential lipid-lowering drugs (Argatroban, Promega, Sulfaphenazole, Sorafenib, Prasterone, Levoxyl, and Alpha-Tocopherol Acetate) were selected, while seven known lipid-lowering drugs with high affinity for target proteins (Apixaban, Implitapide, Tegaserod, Cerivastatin, Etodolac, D-thyroxine, and CES1) were utilized as the positive control group. OpenBabel software (http://openbabel.sf.net) was employed to convert the drug small molecule files from Structure Data File to Protein Data Bank (PDB) format. Subsequently, both the drug ligands and target proteins were preprocessed. For ligands, AutoDockFR processing was performed to obtain PDB, Partial Charge (Q), & Atom Type (T) files of the small molecules. For proteins, crystallographic ligands and water molecules were first removed using PyMOL, followed by ADFR processing to eliminate residual crystallographic water and bound small molecules. Based on these preparations, AutoDock Tools [51] and AutoDock Vina software were utilized to select appropriate docking sites and parameters, followed by the execution of docking calculations. The binding capacity of drug molecules that demonstrated significant lipid-lowering effects in animal experiments was evaluated against these target proteins using AutoDock for semi-flexible docking. A binding energy threshold of −5 kJ/mol was established to determine the affinity levels of positive and candidate drugs for the same receptor protein. Lower binding energy is indicative of stronger binding capacity between the drug molecule and the target protein, implying that the drug may exert its lipid-lowering effect through this receptor protein. Multiple possible conformations for the binding of each drug molecule to the target proteins were generated through molecular docking. The interaction modes between drug molecules and proteins in the conformations with the lowest binding energy were analyzed utilizing PLIP (2021) and LigPlot (Version 2.2.8) [52]. Subsequently, key results were visualized employing PyMOL (Version 2.6) [53].

### Molecular dynamics simulation

Molecular dynamics simulation is a sophisticated computational method widely utilized across engineering and scientific disciplines to calculate the motion and equilibrium states of individual molecules, thus offering detailed insights into complex protein-ligand interactions at atomic resolution and with high temporal precision. In this study, we employ the molecular dynamics simulation software GROMACS 2023 [54], which offers compatibility with various force fields and solvation models. Given AMBER14SB’s proven capability in optimizing protein structures and its appropriateness for simulating macromolecular systems, we employ the AMBER14SB force field in conjunction with the TIP3P water model to perform unconstrained molecular dynamics simulations on the docked complexes of Sorafenib, Sulfaphenazole, Prasterone, Promega, and Argatroban with HMGR, HTR2C, RXRA, MTP, and FX, respectively. Given that the AMBER14SB force field lacks atomic parameters and molecular topologies for the five small molecules under investigation, we utilize the GAFF force field to generate molecular topology files for Metolazone and the other four small molecules that are compatible with the AMBER14SB force field. All simulation systems utilize cubic solvation boxes with periodic boundary conditions applied over a 1 ns timeframe. The system is initially stabilized through 100 ps of NVT (constant Number of particles, Volume, and Temperature) and 100 ps of NPT (constant Number of particles, Pressure, and Temperature) equilibration. Throughout the NVT and NPT ensemble simulations, we employ the V-rescale thermostat coupling algorithm and Parrinello-Rahman pressure coupling method to maintain the system temperature at 300 K and pressure at 1.0 bar, respectively. Subsequently, a 100 ns molecular dynamics simulation of the complex is conducted. We set the non-bonded interaction cut-off value to 1.0 nm and employ the Particle Mesh Ewald method to calculate long-range electrostatic interactions (EEL) at a Coulomb radius of 1.0 nm. We employ a time step of 2 fs and record system conformations every 1000 steps (equivalent to 2 ps). We implement modified Berendsen temperature coupling, setting target temperatures of 300 K for both the complex and water, with a coupling time constant of 0.1 ps. For pressure coupling, we utilize the Parrinello-Rahman algorithm, setting a target pressure of 1.0 bar and a coupling time constant of 2.0 ps.

### Statistical analysis

Statistical analysis and data visualization in this study were performed using R (Version 4.3.0) and Python (Version 3.12.0). For paired samples, we employed the paired Wilcoxon signed-rank test to assess statistical significance. For independent samples, we utilized the Mann–Whitney U test to compare group differences [55]. We utilized the ComplexHeatmap package for generating heatmaps, the circlize package for creating bar plots, ggplot2 for producing box plots and violin plots, and ggplot2 in conjunction with ggalt for constructing dumbbell plots. Statistical significance was defined as a two-sided *P* < 0.05. The following notation was used to indicate significance levels: *P < 0.05, **P < 0.01, ***P < 0.001, and ****P < 0.0001.

## Results

### Machine learning-based identification of lipid-lowering drug candidates

**Fig. 1:**
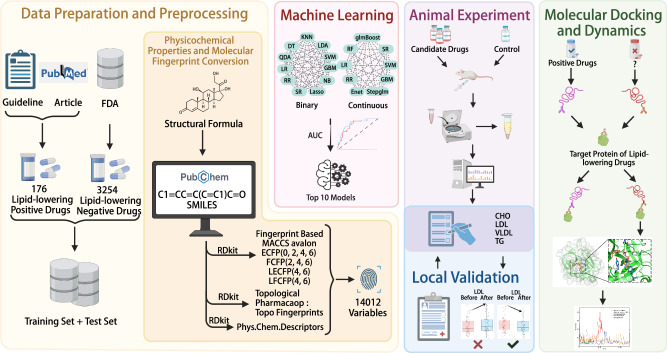
Schematic overview of the development and validation of the machine learning model for predicting the lipid-lowering effect of non-lipid-lowering drugs. This figure was created based on the tools provided by Biorender.com (accessed on 8/2/2024).

**Fig. 2:**
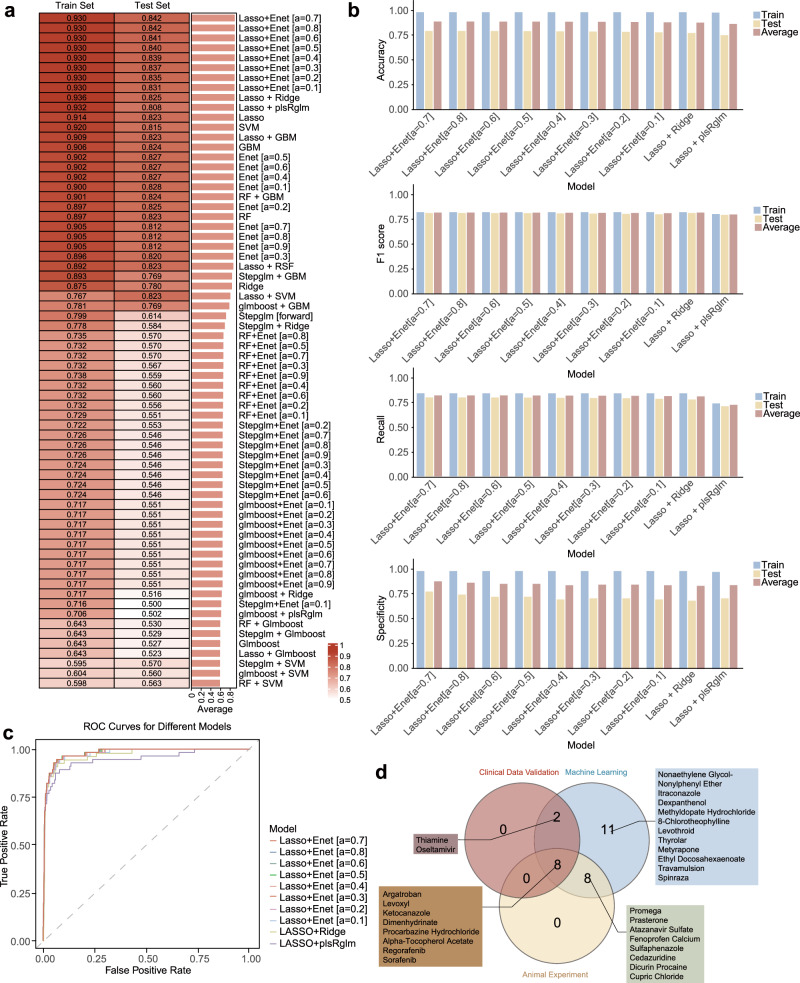
Identification of potential lipid-lowering agents from repurposed drugs using machine learning models. **a** Detailed AUC of the continuous variable machine learning models presented as a heatmap. **b** Evaluation metrics—accuracy, F1 score, recall, and specificity—of the top 10 machine learning models. **c** The ROC curve illustrates the performance of the top ten continuous variable machine learning models. **d** The Venn diagram summarizes the results of repurposed drugs across the continuous variable machine learning model, clinical retrospective data analysis, and animal experiments. In the heatmap, the intensity of the red color represents the magnitude of the corresponding evaluation metrics. Darker shades indicate higher values.

Figure 1 illustrates the comprehensive workflow of this study. Utilizing a dataset comprising 3430 drugs (176 positive drugs with established lipid-lowering effects and 3254 negative drugs), along with their corresponding drug characteristics and lipid-lowering evidence levels, we evaluated the predictive capabilities of various machine learning models. These models incorporated 68 continuous variables (or combinations thereof) to assess the lipid-lowering potential of drugs (Fig. 2a, b). Among the models utilizing continuous variables, the Lasso + Ridge model and the Lasso + Enet model, with various parameter configurations, exhibited exceptional performance. When the regularization parameter α was set to 0.7 for the Lasso + Elastic Net model, it achieved the highest scores in AUC (0.886), accuracy (0.888), F1 score (0.820), recall (0.820), and specificity (0.888), ranking first among all models. Similarly, the Lasso + Partial Least Squares Regression (plsRglm) model, SVM model, Lasso model, and Lasso + GBM model all demonstrated consistently high performance across these five metrics (Fig. 2a, b). Subsequently, we selected the top 10 machine learning models based on their AUC values, which were deemed to have the most robust predictive performance. These models were incorporated into further analyses (Fig. 2c). We further analyzed the lipid-lowering potential assessment results of candidate drugs using the top-performing 10 machine learning models. Analysis of the machine learning results for continuous variables revealed that 29 FDA-approved drugs without lipid-lowering indications were identified as having lipid-lowering potential by at least 8 models (Fig. 2d, Table S7).

In summary, our comprehensive machine learning approach effectively identified 29 FDA-approved drugs with potential lipid-lowering effects, thereby providing a reliable foundation for drug repurposing and subsequent experimental validation.

### Validation of potential lipid-lowering drugs through retrospective clinical data analysis

**Fig. 3:**
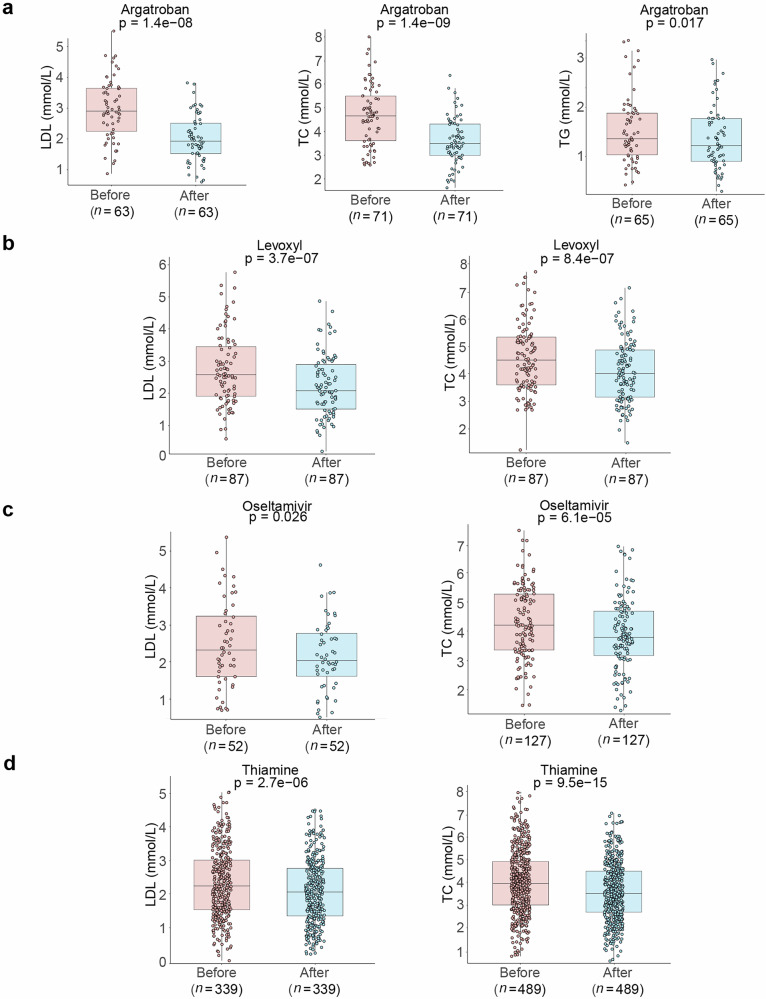
Analysis of differences in TG, TC, HDL, and LDL levels before and after treatment with candidate lipid-lowering drugs based on retrospective clinical data. **a** Box plots depicting the changes in LDL, TC, and TG levels in patients before and after treatment with Argatroban. **b** Box plots depicting the changes in LDL and TC levels in patients before and after treatment with Levoxyl. **c** Box plots depicting the changes in LDL and TC levels in patients before and after treatment with Oseltamivir. **d** Box plots depicting the changes in LDL and TC levels in patients before and after treatment with Thiamine. The corresponding sample sizes are provided. Statistical significance was assessed using the Wilcoxon test.

Comparative analysis of patients’ average blood lipid profiles before and after medication revealed that four drugs (Argatroban, Levoxyl, Oseltamivir, and Thiamine), identified through machine learning screening, exhibited significant biological activity in modulating patients’ blood lipid parameters (Fig. 3a–d). Among these, Argatroban treatment demonstrated the most pronounced effects on blood lipid-related parameters, including low-density lipoprotein (LDL), TC, and TG (Fig. 3a). Analysis of LDL data from 63 patients undergoing Argatroban treatment revealed a significant decrease in LDL levels by 33.1%, from a pre-treatment average of 2.96 mmol/L to 1.98 mmol/L post-treatment (*P* = 1.4 × 10^−8^). Analogously, blood TC and TG levels exhibited significant reductions following medication: TC decreased markedly by 25.1% from a pre-treatment level of 4.68 mmol/L to 3.51 mmol/L post-treatment (*P* = 1.4 × 10^−9^), while TG levels declined from 1.47 mmol/L to 1.37 mmol/L (*P* = 0.017). Levoxyl also exhibited potent lipid-lowering effects (Fig. 3b). Following Levoxyl treatment, 87 patients exhibited significant reductions in both LDL and TC levels, with decreases of 16.2% (*P* = 3.7 × 10^−7^) and 11.9% (*P* = 8.4 × 10^−7^), respectively. Oseltamivir treatment resulted in a reduction of LDL levels and, despite the modest magnitude of change, demonstrated a statistically significant effect on TC reduction in a larger sample size (Fig. 3c). Lastly, Thiamine treatment demonstrated significant lipid-lowering potential, exhibiting notable effects in reducing patients’ LDL and TC levels (Fig. 3d).

In conclusion, the four potential lipid-lowering agents (Argatroban, Levoxyl, Oseltamivir, and Thiamine) identified in this study exhibited significant lipid-modulating effects as evidenced by preliminary clinical data validation. Of particular note, Argatroban demonstrated remarkably pronounced effects in reducing LDL-C, TC, and TG levels while concomitantly elevating HDL-C levels. This observation indicates a high degree of concordance between the predictions generated by the machine learning model and the observed clinical data. However, it is imperative to note that these agents exhibit variations in terms of potency and target specificity, which provides crucial evidence for the development of personalized therapeutic strategies.

### Comprehensive mouse studies validated potential lipid-lowering drug effects

**Fig. 4:**
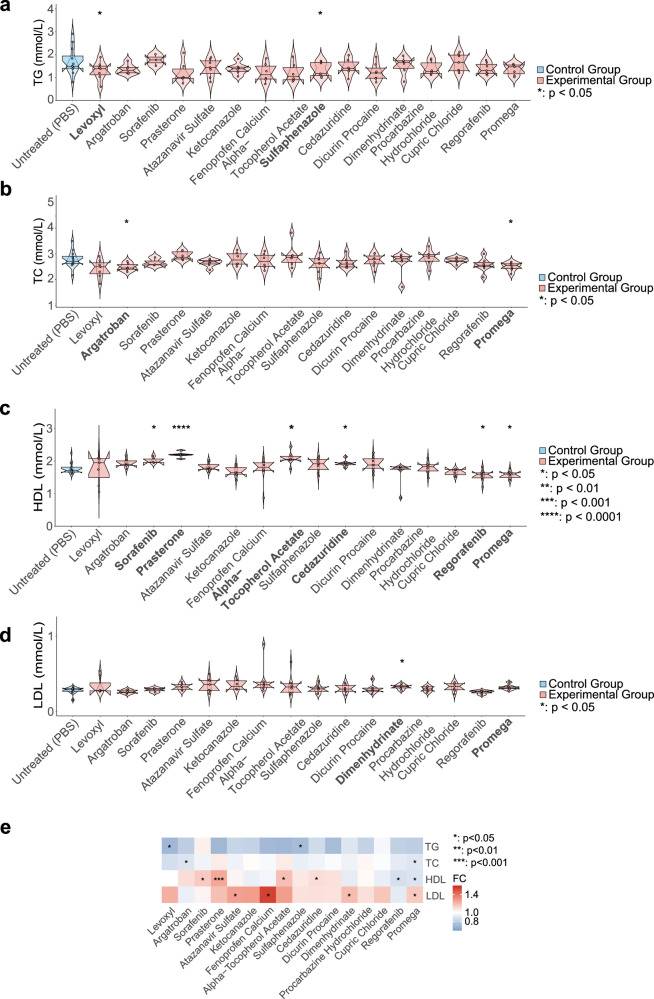
Differences in TG, TC, HDL, and LDL levels before and after treatment with candidate lipid-lowering drugs in an in vivo mouse model. **a** Box plots illustrating TG levels in the experimental group treated with candidate lipid-lowering drugs compared to the PBS control group. **b** Box plots illustrating TC levels in the experimental group versus the PBS control group. **c** Box plots illustrating HDL levels in the experimental group compared to the PBS control group. **d** Box plots illustrating LDL levels in the experimental group versus the PBS control group. **e** The heatmap summarizes the effects of all candidate drugs on mouse blood levels of TG, TC, HDL, and LDL. Each group has a sample size of at least three, with specific sample sizes indicated by points on the box plots. Bold font indicates drugs that resulted in statistically significant changes in lipid levels. Statistical significance was assessed using the Wilcoxon test. *P < 0.05, **P < 0.01, ***P < 0.001, ****P < 0.0001.

In vivo experiments conducted in mouse models demonstrated that multiple drugs significantly modulated four key lipid-related blood indicators: TG, TC, high-density lipoprotein (HDL), and LDL (Fig. 4a–e). Both Levoxyl and Sulfaphenazole exhibited significant TG-lowering effects (*P* < 0.05). Compared to the control group, the Levoxyl treatment group showed a 28.96% reduction in TG levels, while the Sulfaphenazole treatment group demonstrated a 27.09% decrease in TG levels (Fig. 4a). With respect to blood TC levels, we found that Argatroban and Promega significantly reduced blood TC levels: Argatroban treatment lowered TC levels by 10.55% (*P* < 0.05), while Promega treatment reduced TC levels by 9.87% (*P* < 0.05), as shown in Fig. 4b. Furthermore, six drugs - Sorafenib, Prasterone, Alpha-Tocopherol Acetate, Cedazuridine, Regorafenib, and Promega - all exhibited significant effects on blood HDL levels (Fig. 4c). Among all candidate drugs, Prasterone notably exhibited the most pronounced HDL-elevating effect. Relative to the control group, mice in the Prasterone treatment group showed a 24.08% increase in HDL levels (*P* < 0.001). Alpha-tocopherol acetate also demonstrated a substantial increase in HDL: the experimental group showed a significant 17.81% elevation in HDL (*P* = 0.02). Following closely were Sorafenib (*P* = 0.03) and Cedazuridine (*P* = 0.03), both of which significantly increased HDL, with elevations of 14.36% and 9.33%, respectively. Mice treated with Regorafenib and Promega exhibited HDL levels of 1.769 and 1.769 mmol/L, respectively, which were significantly higher than the control group’s 1.593 mmol/L (*P* < 0.05). Contrary to expectations, we found that mice receiving potential lipid-lowering drug treatments had higher LDL levels compared to the control group. LDL levels in the Procarbazine Hydrochloride and Dimenhydrinate treatment groups were both 18.73% higher than those in the control group (*P* = 0.01). The Promega treatment group had an average LDL value of 0.292 mmol/L, representing a 15.19% increase compared to the control group (*P* = 0.04).

In conclusion, this study identified a series of drugs with significant regulatory effects on lipid metabolism in mice through in vivo pharmacological evaluation, with Argatroban, Prasterone, Promega, Sorafenib, and Sulfaphenazole demonstrating particularly pronounced improvements in lipid profile indicators.

### Molecular docking analysis reveals potential targets for lipid-lowering drug action

**Fig. 5:**
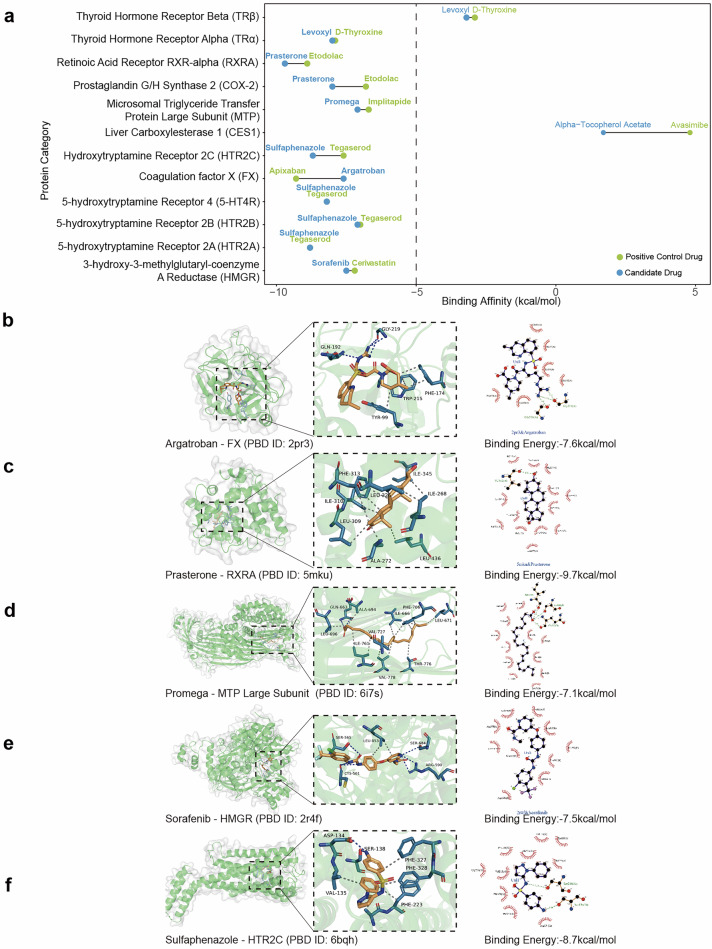
Prediction of binding interactions between candidate lipid-lowering drugs and key lipid metabolism-related target proteins based on molecular docking experiments. **a** The dumbbell chart summarizes the binding affinity of six candidate lipid-lowering drugs with twelve common lipid metabolism-related target proteins. **b** Molecular docking visualization predicting the interaction between Argatroban and Coagulation Factor X (FX). **c** Molecular docking visualization predicting the interaction between Prasterone and Retinoic Acid Receptor RXR-alpha (RXRA). **d** Molecular docking visualization predicting the interaction between Promega and Microsomal Triglyceride Transfer Protein Large Subunit (MTP). **e** Molecular docking visualization predicting the interaction between Sorafenib and 3-Hydroxy-3-Methylglutaryl-Coenzyme A Reductase (HMGR). **f** Molecular docking visualization predicting the interaction between Sulfaphenazole and Hydroxytryptamine Receptor 2 C (HTR2C). A lower binding energy indicates a stronger binding affinity between the drug molecules and target proteins. Bold font indicates that the binding energy of the drug with the corresponding protein is less than −5, signifying a strong interaction.

In this study, we selected seven drugs (Argatroban, Promega, Sulfaphenazole, Sorafenib, Prasterone, Levoxyl, and Alpha-Tocopherol Acetate) that have previously demonstrated lipid-lowering effects in animal experiments and clinical retrospective studies. These drugs were subjected to molecular docking analysis with 12 key target proteins involved in lipid metabolism. Through the evaluation of binding affinities between drugs and target molecules, we investigated the potential lipid-lowering mechanisms of drugs not primarily designed for lipid reduction. The results, as illustrated in Fig. 5a, demonstrate that Argatroban and Apixaban exhibited strong binding affinities to FX, with binding energies of −7.60 and −9.30 kcal/mol, respectively. Promega and Implitapide displayed comparable binding affinities to the MTP, with binding energies of −7.10 and −6.70 kcal/mol, respectively. Sulfaphenazole and Tegaserod exhibited potent binding affinities to serotonin receptors HTR2A, HTR2B, HTR2C, and 5-HT4R, with binding energies consistently below −7.00 kcal/mol. Notably, Sulfadiazine demonstrated the highest binding affinity to HTR2A and HTR2C receptor subtypes, with binding energies ranging from −8.70 to −8.80 kcal/mol. Sorafenib and cerivastatin exhibited robust binding affinities to HMGR, with binding energies of −7.50 and −7.20 kcal/mol, respectively. Prasterone displayed a notable binding affinity to COX-2, with a binding energy of −8.00 kcal/mol, whereas Etodolac exhibited a binding energy of −6.80 kcal/mol to the same target. Additionally, Prasterone and Etodolac showed strong binding affinities to RXRA, with binding energies of −9.70 and −8.90 kcal/mol, respectively. Levoxyl and D-Thyroxine demonstrated strong binding affinities to TRα, with binding energies ranging from −7.90 to −8.00 kcal/mol, while exhibiting weaker affinities to TRβ, as shown in Fig. 5a. Employing molecular docking techniques, we comprehensively evaluated the binding affinities of 12 drug molecules with potential lipid-lowering effects on various lipid metabolism-related targets. Our analysis revealed that multiple drug-target pairs exhibited significant binding affinities, suggesting potential mechanisms for their lipid-lowering actions.

To further elucidate the molecular mechanisms, we conducted comprehensive molecular docking analyses for multiple drugs exhibiting high binding affinity to lipid metabolism-associated target proteins to demonstrate the binding patterns and interactions between drug molecules and target protein sites, including Argatroban with FX, Prasterone with RXRA, Promega with MTP, Sorafenib with HMGR, and Sulfaphenazole with HTR2C (Fig. 5b–f). Molecular docking analyses revealed that Argatroban exhibits high-affinity binding to the active site of FX, establishing multiple crucial interactions with key amino acid residues. In particular, Argatroban establishes hydrophobic interactions with Tyr99A, Phe174A, and Trp215A of FX, simultaneously forming hydrogen bonds with Gln192A and Gly219A of FX, providing additional binding capacity, thereby tightly filling multiple sub-pockets of the thrombin active site and firmly anchoring in the thrombin active center (Fig. 5b). Structural analysis demonstrated that Prasterone establishes extensive hydrophobic interactions with RXRA’s Ile268A, Ala272A, Leu309A, Ile310A, Phe313A, Leu326A, Ile345A, and Leu436A. These comprehensive interactions facilitate Prasterone’s stable accommodation within the RXRA ligand-binding domain. Promega demonstrates dual binding mechanisms, comprising hydrophobic interactions with MTP’s Ile666H, Leu671H, Ala694H, Leu696H, Phe706H, Val727H, Ile761H, Thr776H, and Val778H and hydrogen bonds with MTP’s Gln663H, which synergistically enhance its binding affinity. The binding mode analysis revealed that Sorafenib displays hydrophobic interactions with HMGR’s Leu853B while forming hydrogen bonds with Cys561B, Ser565B, Arg590A, and Ser684A, resulting in tight binding between Sorafenib and HMGR. Sulfaphenazole establishes a network of hydrophobic interactions with HTR2C’s Val135A, Ala222A, Phe223A, Phe327A, and Phe328A, while forming hydrogen bonds with Asp134A and Ser138A, contributing to its strong binding affinity to HTR2C.

### Enhanced exploration of drug-protein binding patterns through molecular dynamics simulations

**Fig. 6:**
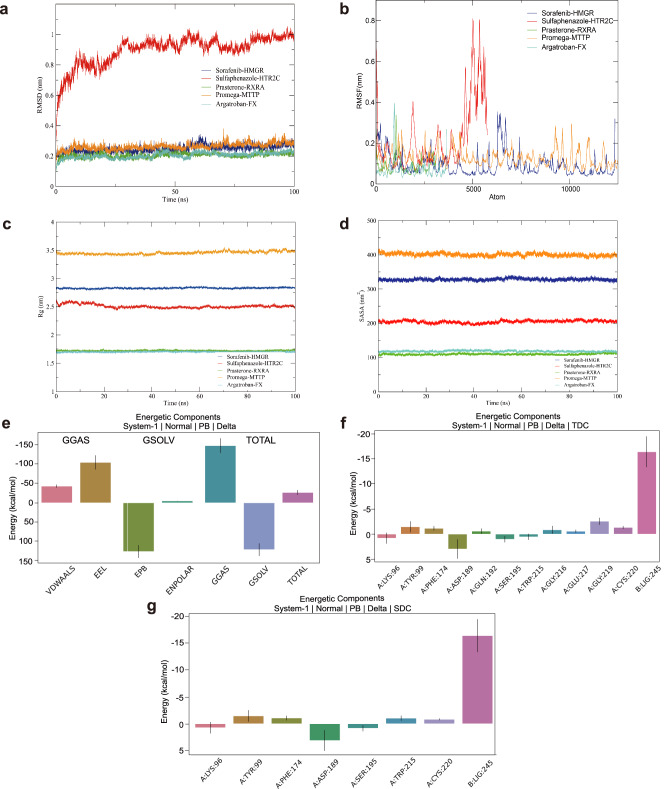
Molecular dynamics simulation and analysis of protein-ligand complexes. **a** Root Mean Square Deviation (RMSD) of the complex. **b** Root Mean Square Fluctuation (RMSF) of the complex. **c** Radius of Gyration (Rg) of the complex. **d** Solvent Accessible Surface Area (SASA) of the complex. **e** MMPBSA analysis of the Argatroban-FX complex. **f** Total Decomposition Contribution (TDC) plot of the Argatroban-FX complex. **g** Sidechain Decomposition Contribution (SDC) plot of the Argatroban-FX complex.

Based on the aforementioned research findings, Sorafenib, Sulfaphenazole, Prasterone, Promega, and Argatroban exhibited significant lipid-lowering effects. To elucidate their mechanisms of action, we conducted an in-depth investigation of these five drugs. Initially, we analyzed the root mean square deviation (RMSD) changes of sulfaphenazole-HTR2C, Sorafenib-HMGR, Prasterone-RXRA, Promega-MTP, and Argatroban-FX complexes over a 100-nanosecond molecular dynamics simulation period. The molecular dynamics simulation results revealed that the sulfaphenazole-HTR2C complex exhibited the highest RMSD value, escalating from 0.3 nm to approximately 1.0 nm, suggesting substantial conformational changes during the ligand-receptor binding process (Fig. 6a). In contrast, the remaining four complexes (Sorafenib-HMGR, Prasterone-RXRA, Promega-MTP, and Argatroban-FX) displayed lower RMSD values, predominantly oscillating between 0.1 and 0.3 nm, indicative of high structural stability (Fig. 6a). Collectively, with the exception of sulfaphenazole-HTR2C, all other complexes demonstrated remarkable structural stability. Root mean square fluctuation (RMSF) analysis of the five ligand-protein complexes indicated that the sulfaphenazole-HTR2C complex displayed the most pronounced fluctuation in the vicinity of 5000 atoms, reaching a peak value of approximately 0.8 nm. Conversely, the RMSF values for the remaining complexes were substantially lower, with the majority of fluctuations not exceeding 0.2 nm. These findings suggest that the sulfaphenazole-HTR2C complex exhibits enhanced flexibility in specific regions, whereas the other complexes maintain relative structural rigidity (Fig. 6b). Analysis of the radius of gyration (Rg) changes for the five ligand-protein complexes during molecular dynamics simulations revealed that the Promega-MTP complex exhibited the highest Rg value of approximately 3.5 nm, followed by Sorafenib-HMGR at 2.8 nm, and sulfaphenazole-HTR2C at 2.5 nm. Prasterone-RXRA and Argatroban-FX displayed the lowest Rg values, both ~1.7 nm (Fig. 6c). The Rg values for all complexes remained relatively constant throughout the simulation period, suggesting that their global conformations did not undergo substantial alterations (Fig. 6c).

The solvent-accessible surface area (SASA) analysis for these five ligand-protein complexes reveals that the Promega-MTP complex demonstrates the largest solvent-exposed area, consistently maintained at approximately 400 nm^2^ (Fig. 6d). The Sorafenib-HMGR and sulfaphenazole-HTR2C complexes exhibit the next highest SASA values, with ~330 nm^2^ and 200 nm^2^, respectively (Fig. 6d). Prasterone-RXRA and Argatroban-FX show smaller solvent contact areas, with values ranging between 110 and 120 nm^2^ (Fig. 6d). The SASA curves for all complexes display relatively stable characteristics, indicating the maintenance of a consistent solvent exposure state throughout the simulation process (Fig. 6d). Furthermore, we performed free energy decomposition analyses for the five protein-ligand systems: Sorafenib-HMGR, sulfaphenazole-HTR2C, Prasterone-RXRA, Promega-MTP, and Argatroban-FX. These analyses included van der Waals forces (VDWAALS), EEL, polar Boltzmann energy (EPB), gas-phase free energy (GGAS), solvation-free energy (GSOLV), and total free energy (TOTAL) (Table 1). The analysis demonstrated that all systems displayed negative total free energies, suggesting thermodynamically favorable interactions (Table 1). Notably, the Promega-MTP system exhibited the lowest total free energy (−43.61 kcal/mol), indicating the strongest binding affinity among the complexes (Table 1). Further analysis showed that VDWAALS significantly contributed to the binding of all systems, while the contribution of EEL varied across systems (Table 1).

**Table 1:** Free energy (kcal/mol) decomposition of the system.

System	VDWAALS	EEL	EPB	GGAS	GSOLV	TOTAL
Sorafenib-HMGR	−33.36	−9.08	24.18	−42.43	20.66	−21.77
Sulfaphenazole-HTR2C	−40.46	−25.19	47.61	−65.65	43.85	−21.8
Prasterone-RXRA	−40.58	−4.79	28.13	−45.37	24.46	−20.91
Promega-MTP	−54.42	−12.94	29.03	−67.37	23.75	−43.61
Argatroban-FX	−43.33	−104.81	126.13	−148.14	121.22	−26.92

Molecular dynamics simulations reveal that Argatroban-FX makes substantial contributions to GGAS, GSOLV, and TOTAL. Within the GGAS component, VDWAALS exhibits a negative value of approximately −50 kcal/mol, while EEL demonstrates a larger negative magnitude of around −100 kcal/mol (Fig. 6e). The GSOLV component comprises EPB, which displays a positive value of approximately 125 kcal/mol, and non-polar solvation-free energy (ENPOLAR), which is marginally positive, approaching zero (Fig. 6e). The TOTAL component analysis indicates that the sum of GGAS exhibits a large negative value of approximately −150 kcal/mol, while the sum of GSOLV is positive, about 120 kcal/mol. Consequently, the final TOTAL is negative, approximately −25 kcal/mol (Fig. 6e). These results suggest that while solvation effects, particularly polar solvation, are detrimental to system stability, gas-phase interactions, notably EEL, contribute more substantially to the system’s stability. The observed negative total energy implies that the molecular system maintains thermodynamic stability under the simulated conditions (Fig. 6e).

**Fig. 7:**
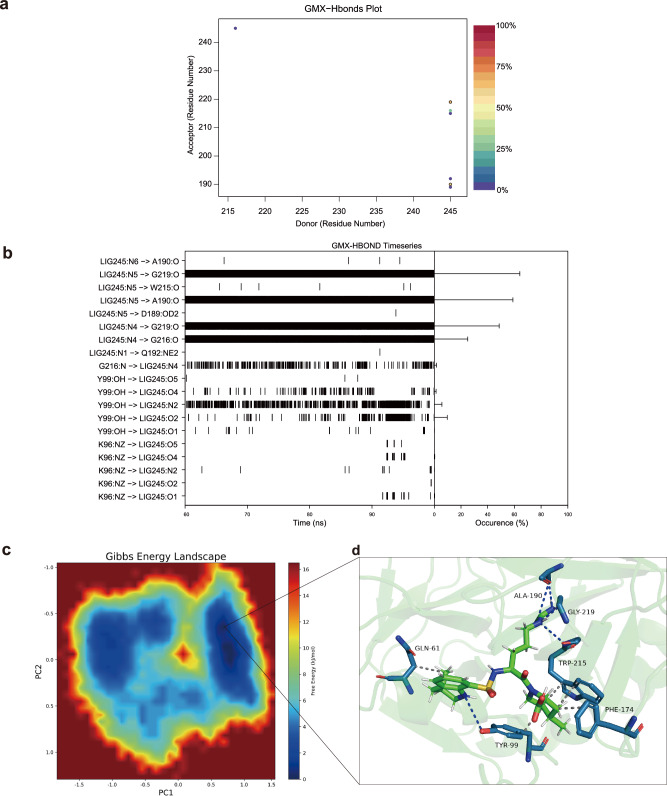
Hydrogen bond analysis and interaction patterns in the Argatroban-FX complex. **a** Hydrogen bond occurrence between donor and acceptor residues in the Argatroban-FX complex. **b** Timeline representation of hydrogen bond formation between different residue pairs in the Argatroban-FX complex. **c** Gibbs Free Energy Landscape of the Argatroban-FX complex obtained from Principal Component Analysis (PCA). **d** Interaction plot of the frame corresponding to the lowest energy in the free energy landscape.

We further calculated the contribution of individual amino acid residues to the total energy in the Total Decomposition Contribution system for the Argatroban-FX complex. The results revealed that the energy contributions of the majority of residues were relatively small, ranging between −1 and 2 kcal/mol (Fig. 6f). Notably, A:GLY:219 and B:LYS:245 exhibited significant positive energy contributions of ~2.5 kcal/mol and 16 kcal/mol, respectively, indicating their potential to generate unfavorable interactions within the system (Fig. 6f). Conversely, A:ASP:189 displayed a notable negative energy contribution of ~ −3 kcal/mol, suggesting its potential crucial role in stabilizing the system structure or promoting favorable interactions (Fig. 6f). In a similar vein, the Sidechain Decomposition Contribution analysis of the Argatroban-FX complex demonstrated that the energy contributions of most amino acid residues to the total energy were relatively small, ranging from −1 to 2 kcal/mol (Fig. 6g). A:ASP:189 exhibited the most significant negative energy contribution of ~ −3 kcal/mol, strongly suggesting its crucial role in stabilizing the system (Fig. 6g). In contrast, LYS245 presented the largest positive energy contribution of ~16 kcal/mol, indicating its potential to generate unfavorable interactions (Fig. 6g). The GMX-Hbonds analysis of the Argatroban-FX complex primarily revealed hydrogen bonds between residues 215 and 245, elucidating key hydrogen bond interactions in the protein structure. These interactions provide valuable insights into protein stability and function (Fig. 7a). Furthermore, the GMX-HBOND time series analysis of the Argatroban-FX complex demonstrated that the hydrogen bonds formed between LIG245 and multiple residues, including G219 and A190, were highly stable, persisting for more than 50% of the entire simulation process. The hydrogen bond between Y99 and LIG245 exhibited relative stability, albeit with intermittent occurrences (Fig. 7b). The interaction between G216 and LIG245 occurred frequently but discontinuously (Fig. 7b). The interaction between K96 and LIG245 showed lower frequency and was predominantly observed in the latter stages of the simulation (Fig. 7b). In the Gibbs free energy landscape of the Argatroban-FX complex, the blue regions denote low-energy states (Fig. 7c), representing the most stable conformations of the complex. We subsequently visualized the molecular interactions within this stable state. The hydrophobic interactions between Argatroban and FX encompassed residues GLN61, TYR99, PHE174, and TRP215, with distances ranging from 3.49 to 3.85 Å, whereas hydrogen bonding interactions involved TYR99 and GLN192, with distances ranging from 2.08 to 2.46 Å. Notably, TYR99 functioned as both a hydrogen bond donor and acceptor, establishing bidirectional interactions with the ligand. GLN192 functioned as a hydrogen bond acceptor. These multiple interactions between Argatroban and FX are likely to contribute substantially to the tight binding observed between the two molecules (Fig. 7c).

## Discussion

This study successfully identified a series of non-traditional drugs with potential lipid-lowering effects by integrating multiple machine learning algorithms. The lipid-lowering efficacy of these drugs was subsequently validated through retrospective clinical data analysis and in vivo experiments in mice. Furthermore, we systematically evaluated the binding capacity and interaction characteristics of these potential lipid-lowering drugs with lipid metabolism-related targets utilizing molecular docking and molecular dynamics simulation techniques. This approach provided a theoretical basis for elucidating their mechanisms of action. The results of the clinical retrospective study validation demonstrated that Argatroban, Levoxyl, Oseltamivir, and Thiamine exhibited significant lipid-regulating effects, thus corroborating the predictions of the machine learning models. In the murine model, drugs including Argatroban, Prasterone, Promega, Sorafenib, and Sulfaphenazole demonstrated notable improvement in blood lipid indicators. From a mechanistic standpoint, Argatroban, Promega, Sulfaphenazole, Sorafenib, Prasterone, and Levoxyl were found to exhibit strong binding affinities to multiple lipid metabolism-related targets, including coagulation factor X, thyroid hormone receptor, and 5-hydroxytryptamine receptor. These findings not only suggest that these drugs may exert lipid-lowering effects through multiple pathways but also offer new insights for developing multi-target lipid-lowering drugs. Molecular docking and molecular dynamics simulation techniques further elucidated the specific binding modes and key interactions between these drugs and their targets, thereby providing important evidence for an in-depth understanding of drug mechanisms and structural optimization. In comparison with existing studies, our approach not only expanded the potential range of lipid-lowering drugs by integrating machine learning with experimental validation but also yielded new insights into drug mechanisms through molecular-level exploration, thus providing a theoretical foundation for developing personalized lipid-lowering treatment strategies.

This study has identified several non-lipid-lowering drugs with significant lipid-lowering potential, among which Argatroban, Levoxyl, and Sulfaphenazole emerged as particularly promising candidates. Although these drugs were originally developed to treat various diseases, our computational model predictions and molecular docking analyses have revealed their potential new mechanisms of action in regulating blood lipids. For instance, Argatroban is clinically employed to treat or prevent heparin-induced thrombocytopenia [56]; however, our machine learning algorithms, clinical data analysis, and animal experiments collectively suggest that Argatroban possesses potential lipid-lowering effects. Patients treated with Argatroban demonstrated significantly reduced levels of LDL, TC, and TG; furthermore, in animal experiments, mice injected with Argatroban also exhibited lower TC levels. Our molecular docking results suggest that Argatroban exhibits a high binding affinity to FX. FX not only plays a crucial role in the coagulation cascade but also modulates adipose tissue inflammation, insulin sensitivity, and fatty acid oxidation through interactions with G protein-coupled receptors, such as protease-activated receptor 2 [57]. Similarly, our study demonstrated that Levoxyl, a standard medication for treating hypothyroidism [58], can significantly improve blood lipid profiles, including LDL, TC, and TG, as corroborated by both clinical retrospective analyses and in vivo animal experimental evidence. Molecular docking analysis revealed that Levoxyl can bind to the TRα subtype with high affinity. These findings suggest that Levoxyl may exert its lipid-lowering effect by modulating the expression of genes involved in lipid metabolism. Notably, while thyroid hormones can accelerate lipolysis and cholesterol metabolism, their lipid-lowering effects are often counteracted by their appetite-stimulating properties [50]. However, Levoxyl exhibits a longer half-life and more stable pharmacokinetic profile compared to endogenous thyroid hormones [59], indicating its potential as a novel long-acting lipid-lowering agent. Furthermore, our research revealed that Sulfaphenazole, an antibiotic primarily used to treat bacterial infections [60], exhibits strong binding affinity to 5-HT2A/2C receptors, indicating its potential to act as a competitive antagonist at serotonin 2A and 2C receptors. Serotonin is known to stimulate the hypothalamus, thereby promoting appetite, while concurrently enhancing lipolysis in adipose tissue [61]. By antagonizing these receptors, Sulfaphenazole may induce appetite suppression, consequently reducing peripheral tissue fat accumulation. These findings not only elucidate potential novel treatment strategies for hyperlipidemia patients but also pave the way for new research directions, facilitating a deeper understanding of the regulatory mechanisms underlying lipid metabolism.

In the realm of lipid-lowering drug development, numerous studies have endeavored to expedite the process of novel drug discovery through the application of computational methods. For example, Rai et al. utilized random forest classifiers to elucidate previously approved drugs with potential lipid-lowering effects [62]. In contrast, our study not only leverages literature support and machine learning predictions but also validates the efficacy of candidate drugs through comprehensive experimental verification, thereby substantially enhancing the credibility and scientific value of the research findings. Furthermore, we have implemented enhancements in data processing and model presentation, significantly augmenting the transparency of data handling and methodology. We have meticulously documented the steps involved in drug-target network construction and analysis, thereby ensuring the reproducibility of the study and facilitating independent verification. Additionally, we have incorporated molecular docking simulations, offering deeper insights into the molecular mechanisms of action of candidate drugs.

Our findings have significant implications for clinical translation. The identified drugs show potential for use in specific patient populations who may not respond well to or tolerate conventional lipid-lowering therapies. Additionally, these drugs might be used in combination with existing lipid-lowering medications to achieve synergistic effects. The molecular mechanisms we uncovered suggest these drugs may regulate lipid metabolism through novel pathways, including potential epigenetic mechanisms. This provides new directions for developing targeted therapies. Furthermore, our integrated machine learning approach combined with multi-omics analysis represents a novel and efficient strategy for drug repositioning that could be applied to other therapeutic areas.

Nevertheless, this study has several notable limitations that warrant consideration. Firstly, the retrospective analysis conducted using local data may not fully account for the potential influence of unknown confounding factors. Consequently, future research should include large-scale, multicenter randomized controlled clinical trials to comprehensively evaluate the lipid-lowering efficacy and long-term safety of these candidate drugs. Secondly, for drug-target pairs exhibiting weak binding affinity, the possibility cannot be discounted that they may exert lipid-lowering effects through alternative mechanisms, or that factors such as receptor structural flexibility may result in positive calculated binding energies. These hypotheses require validation through further biochemical and structural biology experiments. Thus, future research necessitates more in-depth and comprehensive studies to elucidate the lipid-lowering efficacy and mechanisms of non-lipid-lowering drugs, thereby expediting the translation of research findings into clinical practice for patient benefit. Additionally, a notable limitation of this study lies in our unified modeling approach for all lipid-lowering drugs. The 176 positive drugs in our dataset exhibit considerable mechanistic diversity, targeting various molecular pathways and biological processes. This heterogeneity might have prevented the identification of specific feature patterns associated with distinct lipid-lowering mechanisms. Future studies could benefit from stratifying these drugs into mechanistic subcategories - such as HMG-CoA reductase inhibitors, cholesterol absorption inhibitors, and PCSK9 inhibitors - and developing independent predictive models for each category. This stratified approach could potentially enhance prediction accuracy and provide more targeted insights into mechanism-specific drug repurposing opportunities. Such refinement could also facilitate the identification of drugs that act through specific desired mechanisms, potentially leading to more precise therapeutic recommendations. Moreover, future studies could leverage geometric deep learning methods to analyze molecular structures directly. While our approach uses traditional descriptors and fingerprints, pretrained geometric neural networks could capture nuanced structural relationships crucial for drug-target interactions [63, 64]. These methods, learning from 3D conformations and chemical graphs, could complement conventional descriptors and improve understanding of features influencing lipid-lowering efficacy [65].

This study employs an innovative approach by integrating machine learning techniques to systematically explore the lipid-lowering potential of non-lipid-lowering drugs, potentially offering novel treatment options for patients with hyperlipidemia. The research methodology encompasses retrospective clinical data analysis and in vivo animal experiments for validation, while also examining the binding and interaction mechanisms between drugs and lipid-lowering targets at the molecular level. This approach may provide alternative options for patients exhibiting poor tolerance or inadequate response to conventional lipid-lowering therapies, thus offering the potential for individualized and precise treatment of hyperlipidemia. Consequently, this research has the potential to enhance patient outcomes, thereby demonstrating substantial academic value and promising clinical applicability.

## Conclusion

This study innovatively combines machine learning, molecular docking, clinical data analysis, and animal experiments to systematically evaluate the lipid-lowering potential of non-lipid-lowering drugs (such as Argatroban, Levoxyl, and Sulfaphenazole) from multiple dimensions, thereby providing scientific evidence for developing novel lipid-lowering strategies. These drugs exhibited superior lipid-lowering effects in both retrospective clinical studies and animal experiments. We further investigated their potential lipid-lowering mechanisms by examining their binding affinities to certain proteins using molecular docking and molecular dynamics simulation techniques. In conclusion, this study demonstrated through multidimensional analysis that these three non-lipid-lowering drugs exhibit the potential in regulating blood lipid levels through their unique molecular mechanisms, including decreasing TG, lowering LDL-C, and increasing HDL-C. These findings provide innovative strategies and scientific evidence for identifying new lipid-lowering indications in marketed drugs, potentially offering more diversified treatment options for patients with hyperlipidemia.

## Supplementary information

Supplementary information

## Data Availability

The drug information was collected from seven authoritative guidelines, including the guideline of Lipid Management in Patients with Endocrine Disorders, Chinese Guidelines for Lipid Management (2023), and ESC/EAS Guidelines for the Management of Dyslipidemias (2019), along with PubMed literature search between January 2014 and January 2024. The physicochemical properties of the drugs were obtained from PubChem (https://pubmed.ncbi.nlm.nih.gov/), ChemSpider (https://www.chemspider.com/), and DrugBank databases (https://go.drugbank.com/). All other data generated and analyzed in this study are available from the corresponding authors upon reasonable request.
