# Supplementary material for: Integration of machine learning and experimental validation reveals new lipid-lowering drug candidates
Source: Acta Pharmacol Sin. 2025 Apr 15;46(9):2547–63. doi: 10.1038/s41401-025-01539-1 (PMC12373879; doi:10.1038/s41401-025-01539-1)
Supplement: Supplementary file 1 — Supplementary information [file 41401_2025_1539_MOESM1_ESM.docx]

**Supporting Information**

Table S1. Lipid-lowering drugs included in machine learning model training

Table S2. Physicochemical properties of the drugs included in the machine learning model

Table S3. Molecular fingerprints of drugs included in machine learning model training

Table S4. Machine learning model

Table S5. Drug information from animal experiments

Table S6. Target proteins and their corresponding PDB ids

Table S7. Drug candidates for machine learning

**Table S1. Lipid-lowering drugs included in machine learning model training**

| **Drugs** | **Molecularformula** |
| --- | --- |
| Lovastatin | C24H36O5 |
| Simvastatin | C25H38O5 |
| Pravastatin | C23H36O7 |
| Pivastatin;Pitavastatin | C25H24FNO4 |
| Fluvastatin | C24H26FNO4 |
| Atorvastatin | C33H35FN2O5 |
| Rosuvastatin | C22H28FN3O6S |
| Flavone | C15H10O2 |
| Ezetimibe | C24H21F2NO3 |
| Inclisiran | C78H140N11O34P |
| Probucol | C31H48O2S2 |
| Cholestyramine | C27H47ClN2 |
| Colesevelam | C31H67Cl3N4O |
| Polydodecanol | C30H62O10 |
| Fenofibrate | C20H21ClO4 |
| Bezafibrate | C19H20ClNO4 |
| Gemfibrozil | C15H22O3 |
| Pemafibrate | C28H30N2O6 |
| Niacinnicotinic Acid | C6H5NO2 |
| Mipomersen | C230H324N67O122P19S19 |
| Lomitapide | C39H37F6N3O2 |
| Bempedoic Acid | C19H36O5 |
| Volanesorsen | C230H320N63O125P19S19 |
| Docosahexaenoic Acid | C14H34I2N4 |
| Icosapent Ethyl | C22H34O2 |
| Eicosapentaenoic Acid ;EPA | C20H30O2 |
| Alpha-linolenic Acid ;ALA | C18H30O2 |
| Docosahexaenoic Acid ;DHA | C22H32O2 |
| Neomycin | C23H46N6O13 |
| D-Thyroxine | C15H11I4NO4 |
| Ciprofibrate | C13H14Cl2O3 |
| Clofibrate | C12H15ClO3 |
| Cerivastatin | C26H34FNO5 |
| Mevastatin | C23H34O5 |
| Avasimibe | C29H43NO4S |
| Alufibrate | C20H22AlCl2O7 |
| KB-141 | C17H16Cl2O4 |
| Rimonabant | C22H21Cl3N4O |
| Orlistat | C29H53NO5 |
| Implitapide | C35H37N3O2 |
| Lapaquistat | C31H39ClN2O8 |
| Epanova | C60H92O6 |
| Hesperetin;Brutieridin | C16H14O6 |
| Lovastatin Acid | C24H38O6 |
| Tocotrienol | C26H38O2 |
| Anacetrapib | C30H25F10NO3 |
| Pradigastat | C25H24F3N3O2 |
| Pamaqueside;CP- 148，623 | C39H62O14 |
| MBX-8025 | C21H23F3O5S |
| Crilvastatin | C14H23NO3 |
| Granotapide | C39H37F3N2O8 |
| Sobetirome;GC-1 | C20H24O4 |
| VIA-3196;Resmetirom | C17H12Cl2N6O4 |
| Eprotirome | C18H17Br2NO5 |
| Evacetrapib | C31H36F6N6O2 |
| DRL-17822 | C30H31F6N7 |
| Eprotirome | C23H35NO2S |
| Evacetrapib | C26H25F9N2O4 |
| RVX-208 | C20H22N2O5 |
| GSK-256073 | C10H13ClN4O2 |
| Darapladib;Varespladib;Rilapladib | C36H38F4N4O2S |
| Aleglitazar | C24H23NO5S |
| ZYH1;Saroglitazar | C25H29NO4S |
| GFT-505;Elafibranor | C22H24O4S |
| PRC-4016;Icosabutate | C24H38O3 |
| Ibrolipim | C19H20BrN2O4P |
| MB-07811 | C28H32ClO5P |
| S-8921 | C30H36O9 |
| SLx-4090 | C31H25F3N2O4 |
| TA-8995;Obicetrapib | C32H31F9N4O5 |
| Axitirome | C25H24FNO6 |
| GS 9667 | C21H24FN5O4S |
| GW409544 | C31H30N2O5 |
| Turofexorate Isopropyl | C25H24F2N2O3 |
| Dalvastatin | C24H31FO3 |
| Lapaquistat Acetate | C33H41ClN2O9 |
| BMS-187745 | C16H19O7PS |
| BMY-21950 | C23H21F2N4NaO4 |
| BMY-22089 | C23H20F2N4O3 |
| CL-283796 | C28H41ClN2O |
| E-5324 | C26H34N4O2 |
| Eflucimibe | C29H43NO2S |
| KRP-101 | C26H26FNO5 |
| KRP-297 | C20H17F3N2O4S |
| RP-64477 | C29H42N2O3S |
| SCH-48461 | C26H27NO3 |
| Tiqueside | C39H64O13 |
| 447C88 | C27H38F2N2O |
| YM-17E | C40H58Cl2N6O2 |
| YM-750 | C31H36N2O |
| Melinamide | C26H41NO |
| FR-194738 | C27H38ClNO2S |
| Dup128 | C34H40F2N4OS |
| SQ-33600 | C23H21FNNa2O5P |
| Naringenin;Melitidin | C34H42O19 |
| Nicotinyl Alcohol | C6H7NO |
| Colestipol | C8H24ClN5 |
| Carnitine | C7H15NO3 |
| Lifibrol | C21H26O4 |
| Acipimox | C6H6N2O3 |
| Muvalaplin | C42H54N4O6 |
| Campesterol | C28H48O |
| Ketoconazole | C26H28Cl2N4O4 |
| Lipostabil | C12H14Cl3O3P |
| Etiroxate | C18H17I4NO4 |
| Laropiprant | C21H19ClFNO4S |
| Tricaprin | C33H62O6 |
| Gemcadiol | C14H30O2 |
| Dextran Sulfate | C9H11NO2 |
| D-Thyroxine ;Dynothel | C15H10I4NNaO4 |
| Halofenate | C19H17ClF3NO4 |
| Xantinol-Nicotinate | C19H26N6O6 |
| Tiadenol | C14H30O2S2 |
| MK-0616 | C81H109FN15O15S2+ |
| Sitostanol | C29H52O |
| Methyldopa | C10H13NO4 |
| Acifran | C12H10O4 |
| Pantethine | C22H42N4O8S2 |
| Policosanol | C3H8O |
| Hybutimibe | C25H21F2NO3 |
| Etofibrate | C18H18ClNO5 |
| Pirifibrate | C17H18ClNO4 |
| Pirozadil | C27H29NO10 |
| Sitosterol | C29H50O |
| SLN360（zerlasiran） | C66H122N6O38P4S4 |
| Lovaza | C46H70O4 |
| Atorvastatin Calcium | C66H68CaF2N4O10 |
| Atorvastatin Calcium Trihydrate | C66H74CaF2N4O13 |
| Rosuvastatin Calcium | C44H54CaF2N6O12S2 |
| Rosuvastatin Zinc | C44H54F2N6O12S2Zn |
| Pravastatin Sodium | C23H35NaO7 |
| Fenofibric Acid | C17H15ClO4 |
| Niacinamide | C6H6N2O |
| Cerivastatin Sodium | C26H33FNNaO5 |
| (S)-2-Methyl-butyric acid (1S，3R，7S，8S，8aR)-8-[2-((R)-4-hydroxy-6-oxo-tetrahydro-pyran-2-yl)-ethyl]-3，7-dimethyl-1，2，3，7，8，8a-hexahydro-naphthalen-1-yl ester | C24H36O5 |
| (+)-(3R，5S)-XU 62-320 (free acid) | C24H25FNNaO4 |
| (3R，5S，E)-7-(2-cyclopropyl-4-(4-fluorophenyl)quinolin-3-yl)-3，5-dihydroxyhept-6-enoate | C25H23FNO4- |
| (9Z，12Z，15Z)-octadeca-9，12，15-trienoic acid | C18H30O2 |
| 2，3-Dihydroxypropyl octanoate | C11H22O4 |
| Abiraterone Acetate | C26H33NO2 |
| Alpha-Tocopherol | C29H50O2 |
| Anastrozole | C17H19N5 |
| Apixaban | C25H25N5O4 |
| Argatroban | C23H36N6O5S |
| Atazanavir | C38H52N6O7 |
| Calcibind | C20H39O25P3 |
| Choline Fenofibrate | C22H28ClNO5 |
| Etodolac | C17H21NO3 |
| Etrasimod | C26H26F3NO3 |
| Etravirine | C20H15BrN6O |
| Evotaz | C78H105N13O12S2 |
| Liptruzet | C90H89CaF4N5O13 |
| Obeticholic Acid | C26H44O4 |
| Pantothenic Acid | C9H17NO5 |
| Pioglitazone | C19H20N2O3S |
| Pitavastatin Calcium | C50H46CaF2N2O8 |
| Pitavastatin Magnesium | C50H46F2MgN2O8 |
| Pitavastatin Sodium | C25H23FNNaO4 |
| Ramipril | C23H32N2O5 |
| Sodium Heparin | C26H41NO34S4 |
| Sorafenib | C21H16ClF3N4O3 |
| Spirapril | C22H30N2O5S2 |
| Sugammadex | C72H112O48S8 |
| Sulfinpyrazone | C23H20N2O3S |
| Sulindac | C20H17FO3S |
| Tadalafil | C22H19N3O4 |
| Tazarotene | C21H21NO2S |
| Tegaserod | C16H23N5O |
| Telmisartan | C33H30N4O2 |
| Thiamine Hydrochloride | C12H18Cl2N4OS |
| Tolazamide | C14H21N3O3S |
| Tolbutamide | C12H18N2O3S |
| Triheptanoin | C24H44O6 |
| Troglitazone | C24H27NO5S |
| Ursodiol | C24H40O4 |
| Vytorin | C49H59F2NO8 |

| **Property** | **The Name of Property** | **Meaning** |
| --- | --- | --- |
| Molecular Weight | MolWt | The sum of the atomic weights of all atoms in a molecule. |
| Heavy Atom Count | HeavyAtomCount | Number of non-hydrogen atoms in a molecule. |
| Number of Hydrogen Bond Acceptors | NumHAcceptors | Atoms which can accept hydrogen bonds; typically oxygen and nitrogen. |
| Number of Hydrogen Bond Donors | NumHDonors | Atoms or groups in the molecule that can donate a hydrogen atom in a bond. |
| Logarithm of the Octanol-Water Partition Coefficient | MolLogP | The logarithm of the compound's partition coefficient between octanol and water. |
| Number of Rotatable Bonds | NumRotatableBonds | Single non-ring bonds， excluding those bound to terminal alkyl groups. |
| Ring System | Ring | A structure consisting of two or more connected rings of atoms. |
| Topological Polar Surface Area | TPSA | A measure of the molecule's polar surface area contributed by oxygen and nitrogen. |
| Calculated Octanol-Water Partition Coefficient | cLogP | Computationally derived logP value estimating the lipophilicity of a compound. |
| Molar Refractivity | MR | Measure of the total polarizability of a molecule. |
| Molecular Polarizability | IPC | Indicates how easily a molecule's electron cloud can be distorted. |
| Number of Specified Stereocenters | Nspecified stereo centers | Chiral centers in a molecule with a defined stereo configuration. |
| Number of Aromatic Rings | NumAromaticRings | Rings in a molecule that exhibit aromaticity. |
| Number of Chiral Centers | Chiral centers | Atoms in a molecule that have four different substituents， leading to isomerism. |
| Number of Stereoisomers | stereoisomers | Possible isomers of a molecule that differ only in the spatial orientation of atoms. |
| Molecular Charge | charge | The net electrical charge of a molecule. |

**Table S2. Physicochemical properties of the drugs included in the machine learning model**

**Table S3. Molecular fingerprints of drugs included in machine learning model training**

| **Fingerprint** | **Meaning** |
| --- | --- |
| MACCS | Molecular ACCess System: A set of 166 predefined structural key bits that represent the presence or absence of particular substructures in a molecule |
| Avalon | A type of fingerprint generated by a hashing algorithm that takes into account atom types， bond types， and molecular topology |
| topo fingerprint | Topological fingerprints: Represent the molecular structure based on the connectivity of atoms without considering the types of atoms or bonds |
| ECFP | A type of circular fingerprint that represents the molecular structure by considering the atom environments iteratively expanded out to a specified diameter |
| FCFP | Similar to ECFP but focuses on functional groups instead of individual atoms， emphasizing the chemical functionality |
| LECFP | An extension of ECFP that adds layers of information from additional iterations over the molecular structure， increasing detail with each layer |
| LFCFP | An extension of FCFP that includes layers of functional class information， providing a richer representation of chemical functionality |

**Table S4. Machine learning model**

| **Model** |
| --- |
| Lasso + Ridge |
| Lasso + plsRglm |
| Lasso + Enet [α=0.1] |
| Lasso + Enet [α=0.3] |
| Lasso + Enet [α=0.4] |
| Lasso + Enet [α=0.5] |
| Lasso + Enet [α=0.2] |
| Lasso + Enet [α=0.6] |
| Lasso + Enet [α=0.7] |
| Lasso + Enet [α=0.8] |
| SVM |
| Lasso |
| Lasso + GBM |
| GBM |
| Enet [α=0.7] |
| Enet [α=0.8] |
| Enet [α=0.9] |
| Enet [α=0.5] |
| Enet [α=0.6] |
| Enet [α=0.4] |
| RF + GBM |
| Enet [α=0.1] |
| RF |
| Enet [α=0.2] |
| Enet [α=0.3] |
| Stepglm + GBM |
| Lasso + RSF |
| Ridge |
| Stepglm [forward] |
| glmboost + GBM |
| Stepglm + Ridge |
| Lasso + SVM |
| RF + Enet [α=0.9] |
| RF + Enet [α=0.8] |
| RF + Enet [α=0.2] |
| RF + Enet [α=0.3] |
| RF + Enet [α=0.4] |
| RF + Enet [α=0.5] |
| RF + Enet [α=0.6] |
| RF + Enet [α=0.7] |
| RF + Enet [α=0.1] |
| Stepglm + Enet [α=0.7] |
| Stepglm + Enet [α=0.8] |
| Stepglm + Enet [α=0.9] |
| Stepglm + Enet [α=0.3] |
| Stepglm + Enet [α=0.4] |
| Stepglm + Enet [α=0.5] |
| Stepglm + Enet [α=0.6] |
| Stepglm + Enet [α=0.2] |
| glmboost + Enet [α=0.1] |
| glmboost + Enet [α=0.2] |
| glmboost + Enet [α=0.3] |
| glmboost + Enet [α=0.4] |
| glmboost + Enet [α=0.5] |
| glmboost + Enet [α=0.6] |
| glmboost + Enet [α=0.7] |
| glmboost + Enet [α=0.8] |
| glmboost + Enet [α=0.9] |
| glmboost + Ridge |
| Stepglm + Enet [α=0.1] |
| glmboost + plsRglm |
| RF + glmboost |
| Lasso + glmboost |
| Stepglm + glmboost |
| glmboost |
| glmboost + SVM |
| RF + SVM |
| Stepglm + SVM |

**Table S5. Drug information from animal experiments**

| **Group** | **Drug** | **Catalog No.** | **Dosage** | **Brand** |
| --- | --- | --- | --- | --- |
| Control Group | PBS |  |  | Servicebio |
| Experimental Groups | Levoxyl | C6460-25 | 100.00μg/kg | APExBIO |
|  | Argatroban | A822301-50mg | 10.00mg/kg | Macklin |
|  | Sorafenib | S47444-50mg | 10.00mg/kg | Yuanye |
|  | Prasterone | SE-MM-CD4222-1g | 10.00mg/kg | SALMART |
|  | Atazanavir Sulfate | A833243-200mg | 10.00mg/kg | Macklin |
|  | Ketocanazole | K822880-1g | 50.00mg/kg | Macklin |
|  | Fenoprofen Calcium | T6497-100 mg | 20.00mg/kg | TargetMol |
|  | Alpha-Tocopherol Acetate | V6207-1g | 100.00mg/kg | InvivoChem |
|  | Sulfaphenazole | S80778-25mg | 5.13mg/kg | Yuanye |
|  | Cedazuridine | 38767-1 | 2.50mg/kg | Cayman |
|  | Dicurin Procaine | R007223-25g | 50.00mg/kg | RHAWN |
|  | Dimenhydrinate | D860018-1g | 60.00mg/kg | Macklin |
|  | Procarbazine Hydrochloride | P859902-1g | 60mg/kg | Macklin |
|  | Cupric Chloride | Y-ZH-11910-1g | 10mg/kg | NEW RESEARCH BIOSCIENCES |
|  | Regorafenib | R873734-5mg | 5mg/kg | Macklin |
|  | Promega | P135003-50mg | 100mg/kg | Aladdin |

**Table S6. Target proteins and their corresponding PDB ids**

| **Protein** | **Pdb Id** |
| --- | --- |
| Liver carboxylesterase 1 | 5a7g |
| Coagulation factor X | 2pr3 |
| Microsomal triglyceride transfer protein large subunit | 6i7s |
| 5-hydroxytryptamine receptor 4 | 7xta E（r>3) |
| hydroxytryptamine receptor 2C | 6bqh |
| 5-hydroxytryptamine receptor 2A | 7WC8_m |
| 5-hydroxytryptamine receptor 2B | 7srq A |
| 3-hydroxy-3-methylglutaryl-coenzyme A reductase | 2r4f_m |
| Prostaglandin G/H synthase 2 | 5ikt |
| Retinoic acid receptor RXR-alpha | 5mku |
| Thyroid hormone receptor alpha | 3ilz |
| Thyroid hormone receptor beta | 6kkb |

**Table S7. Drug candidates for machine learning**

| **Drug** | **Positive Counts** |
| --- | --- |
| Argatroban monohydrate | 10 |
| Prasterone | 10 |
| Levothroid | 10 |
| Levoxyl | 10 |
| THYROLAR | 10 |
| Ketocanazole | 10 |
| Atazanavir Sulfate | 10 |
| Metyrapone | 10 |
| (6E)-7-[3-(4-fluorophenyl)-1-(propan-2-yl)-1H-indol-2-yl]-3,5-dihydroxyhept-6-enoic acid | 10 |
| Fenoprofen Calcium | 10 |
| Alpha-Tocopherol Acetate | 10 |
| Sorafenib Tosylate | 10 |
| Spinraza | 10 |
| Promega | 10 |
| Nonaethylene glycol nonylphenyl ether | 10 |
| TRAVAMULSION | 10 |
| Regorafenib | 10 |
| Sulfaphenazole | 9 |
| Cedazuridine | 9 |
| 1-[4-[4-[[2-(2,4-Dichlorophenyl)-2-(imidazol-1-ylmethyl)-1,3-dioxolan-4-yl]methoxy]phenyl]piperazin-1-yl]ethanone | 9 |
| Dexpanthenol | 9 |
| DICURIN PROCAINE | 9 |
| Methyldopate Hydrochloride | 9 |
| Cupric Chloride | 9 |
| 8-Chlorotheophylline | 9 |
| Dimenhydrinate | 9 |
| Procarbazine Hydrochloride | 8 |
| Thiamine | 8 |
| Oseltamivir | 8 |
